# A case report of crystalline light chain inclusion-associated kidney disease affecting podocytes but without Fanconi syndrome: A clonal analysis of pathological monoclonal light chain

**DOI:** 10.1097/MD.0000000000013915

**Published:** 2019-02-01

**Authors:** Kiyoaki Ito, Satoshi Hara, Kazunori Yamada, Takeshi Zoshima, Ichiro Mizushima, Hiroshi Fujii, Ryoichi Miyazaki, Yasukazu Kawai, Akihiro Yachie, Michio Nagata, Shozo Izui, Masakazu Yamagishi, Mitsuhiro Kawano

**Affiliations:** a Division of Rheumatology, Department of Internal Medicine, Kanazawa University Graduate School of Medicine, Kanazawa; b Department of Internal Medicine, Fujita Memorial Hospital; c Department of Hematology and Oncology, Fukui Prefectural Hospital, Fukui; d Department of Pediatrics, Kanazawa University Graduate School of Medicine, Kanazawa; e Department of Kidney and Vascular Pathology, Faculty of Medicine, University of Tsukuba, Tsukuba, Japan.; f Department of Pathology and Immunology, University Medical Center, University of Geneva, Switzerland; g Division of Cardiology, Department of Internal Medicine, Kanazawa University Graduate School of Medicine, Kanazawa, Japan.

**Keywords:** crystal-storing histiocytosis, light chain, light chain proximal tubulopathy, podocyte

## Abstract

**Rationale:**

Crystalline light chain inclusion-associated kidney disease affects mainly tubular epithelial cells and is often clinically manifested as Fanconi syndrome. However, only very few case reports about the crystalline deposits within the podocytes are available, and the nature of the pathogenic monoclonal light chain implicated in these cases is still unknown. We report a case of crystalline inclusion-associated kidney disease manifested as crystalline podocytopathy in which we identified the complete structure of the pathogenic monoclonal light chain as belonging to the germ-line gene of Vκ1-39.

**Patient concerns:**

We describe a 65-year-old woman with crystalline light chain inclusion-associated kidney disease showing mild proteinuria and renal insufficiency with monoclonal gammopathy of undetermined significance without Fanconi syndrome. She had crystalline inclusions mainly within podocytes, tubular epithelial cells and histiocytes in the kidney. Light microscopy showed vacuolation of podocytes and tubular epithelial cells, while eosin negative pale needle-like crystals were present within these cells. Electron microscopy showed accumulation of club-like crystals with high electron density in podocytes, proximal tubular epithelial cells and interstitial histiocytes. Clonal analysis revealed that a pathogenic monoclonal light chain was derived from germline gene, Vκ1-39.

**Diagnoses:**

The diagnosis of crystalline light chain inclusion-associated kidney disease was made.

**Interventions and outcomes:**

Bortezomib and dexamethasone were started and her renal function improved to eGFR 36 mL/min/1.73 m^2^ after 9 courses of therapy.

**Lessons:**

Patients with light chain crystalline podocytopathy may have a similar pathogenic monoclonal light chain derived from the same germline gene, Vκ1–39, to that of patients with light chain proximal tubulopathy.

## 1 Introduction

Plasma cell dyscrasia often induces renal dysfunction associated with monoclonal immunoglobulin. In most cases, monoclonal immunoglobulin accumulates extracellularly as casts, fibers, or granules resulting in cast nephropathy, amyloidosis, or monoclonal immunoglobulin deposition disease.^[1]^ On the other hand, although very rare, the entity of crystalline light chain inclusion-associated kidney disease in which monoclonal light chain crystalline accumulates within cells also exists.^[2]^ In this condition, crystalline inclusions form mainly within the tubular epithelial cells. This condition is referred to as light chain proximal tubulopathy (LCPT), and often induces Fanconi syndrome clinically.^[3,4]^ Interestingly, light chain crystalline deposits can be formed within the podocytes.^[5–15]^ In such cases, not Fanconi syndrome but proteinuria, nephrotic syndrome, or renal insufficiency are the main clinical manifestations. However, only very few case reports about the crystalline deposits within the podocytes are available, and the nature of the pathogenic monoclonal light chain implicated in these cases is still unknown. Here, we report a case of crystalline inclusion-associated kidney disease manifested as crystalline podocytopathy in which we identified the complete structure of the pathogenic monoclonal light chain as belonging to the germ-line gene of Vκ1-39.

## 2 Case

A 65-year-old Japanese woman was admitted to our hospital for close examination of decreased renal function. Her father had cardiac disease and her mother had liver cirrhosis. When she developed maxillary sinusitis 3 years before, renal insufficiency was pointed out with a serum creatinine level (sCr) of 1.15 mg/dL. One year before admission, her sCr rose to 1.31 mg/dL and urine protein was 2.0 g/gCr, but kidney biopsy revealed interstitial fibrosis and tubular atrophy without any glomerular abnormalities, and the etiology could not be determined. Although she had been treated with valsartan, her sCr gradually worsened to 1.94 mg/dL.

On admission, her blood pressure was 110/60 mmHg, pulse rate was 84/min, and body temperature was 36.2°C. The heart, lungs, and abdominal findings were normal. No lower leg edema, skin rashes, or neurological abnormalities were noted. Urinalysis showed 2+ proteinuria, 2+ occult blood, and no glycosuria. Urinary sediment showed neither granular nor red blood cell casts. Proteinuria was 1.31 g/gCr. Blood examination revealed renal insufficiency [Cr 1.94 mg/dL, estimated glomerular filtration rate (eGFR) 21.0 mL/min/1.73 m^2^]. No evidence of Fanconi syndrome was noted (uric acid 3.0 mg/dL, sodium 139 mEq/L, potassium 3.9 mEq/L, calcium 8.8 mg/dL, phosphate 3.3 mg/dL). Her immunological data showed normal complement levels [C3 108 mg/dL (normal: 44–102 mg/dL), CH50 40 U/mL (normal: 32–47 U/mL)] but mild elevation of immunoglobulin G (IgG) with decreased levels of IgA and IgM (IgG 2,259 mg/dL, IgA 47 mg/dL, IgM 42 mg/dL). Anti-nuclear antibodies were negative. Immunoelectrophoresis revealed serum monoclonal IgG κ and urine κ Bence-Jones protein. Because bone marrow biopsy showed a plasma cell fraction of less than 10%, the diagnosis of monoclonal gammopathy of undetermined significance (MGUS) was made.

**Figure 1:**
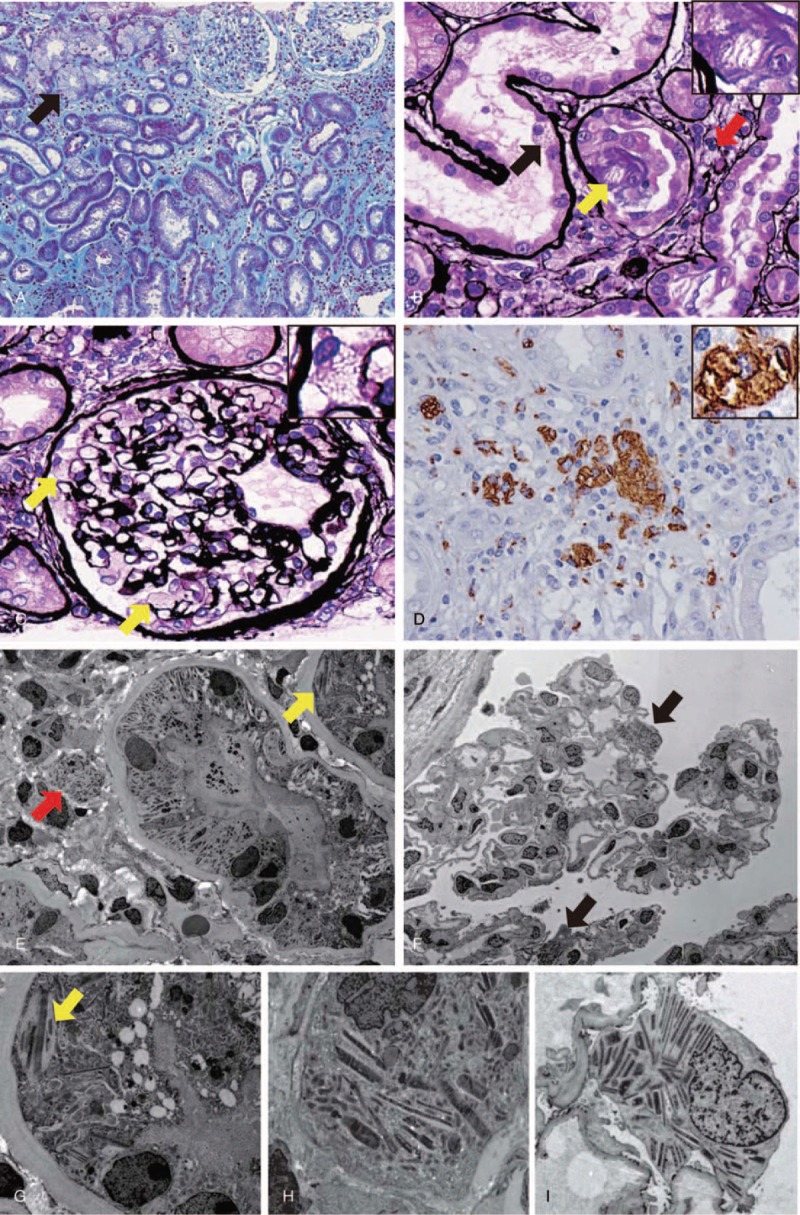
Light microscopy and electron microscopy findings of the patient. (A) Tubulointerstitium showed diffuse interstitial fibrosis. Some proximal and distal tubular epithelial cells were vacuolated (arrow) (Masson-Trichrome staining). (B) Eosin-negative pale needle-like crystals were present both in tubular epithelial cells (black arrow) and interstitium (red arrow). Note that denuded cell contains the crystals in the tubular lumen (yellow arrow) (Periodic acid-Schiff methenamine silver stain). (C) The glomeruli showed vacuolation of podocytes (arrows) without mesangial proliferation, endocapillary proliferation, or extracapillary proliferation (Periodic acid-Schiff methenamine silver stain). (D) CD68 immunostaining revealed the presence of needle- or polygon-like crystals in CD68-positive macrophages. (E, G, H) Electron microscopy showed accumulation of club-like crystals with high electron density in proximal tubular epithelial cells (yellow arrows, G) and interstitial histiocytes (red arrow, H). (F, I) A similar accumulation of club-like crystals with high electron density was present in podocytes (arrows, I). (Original magnification, A, ×100, C, ×200, B, D, ×400, E, ×600, F, ×400, G, ×6000, H, ×7000, I, ×5000).

**Figure 2:**
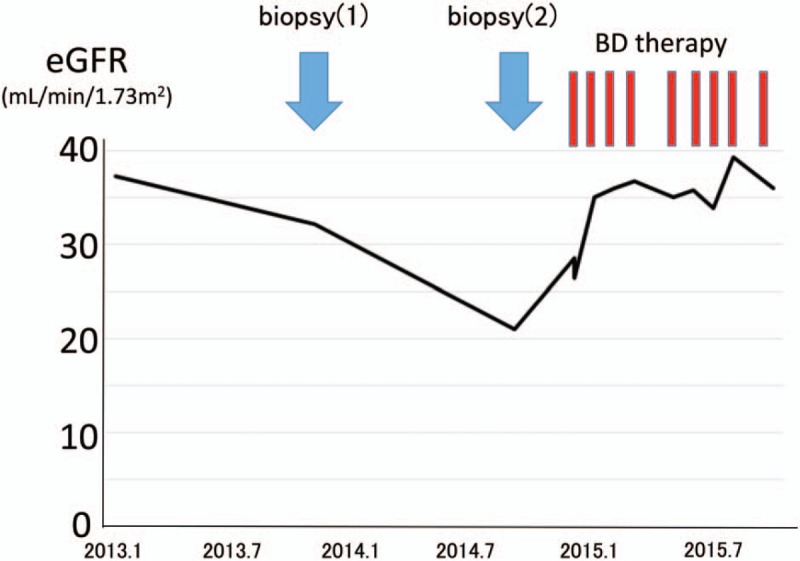
Clinical course. Her renal function improved after 9 courses of therapy. BD = Bortezomib and dexamethasone.

On admission, a kidney biopsy was performed. The renal specimens contained 17 glomeruli, 7 of which showed global sclerosis. The tubulointerstitium showed diffuse interstitial fibrosis with some inflammatory cell infiltration (Fig. 1A). A substantial number of proximal and distal tubular epithelial cells had a foamy appearance (Fig. 1A–B) which consisted of eosin- and trichrome-negative pale needle-like crystals (Fig. 1B). These crystals were also present in the interstitium and glomerular podocytes, showing a foamy appearance (Fig. 1B–C). The glomeruli showed minor abnormalities (Fig. 1C). Immunostaining revealed CD68-positive macrophages containing the needle-like crystals in the interstitium (Fig. 1D). Immunofluorescence showed negative staining of IgG, IgA, IgM, C3, C1q, κ, and λ. Electron microscopy showed accumulation of club-like crystals with high electron density in proximal tubular epithelial cells (PTE) and interstitial histiocytes (Fig. 1E–G). A similar accumulation of club-like crystals with high electron density was present in podocytes (Fig. 1F–H). The diagnosis of crystalline light chain inclusion-associated kidney disease was made. Bortezomib and dexamethasone were started and her renal function improved to eGFR 36 mL/min/1.73 m^2^ after 9 courses of therapy (Fig. 2).

## 3 Clonal analysis

The nucleotide sequence corresponding to the variable and constant regions of pathogenic monoclonal kappa light chain was determined using kappa specific primers and cDNAs synthesized on a template of RNA extracted from bone marrow as previously described.^[16,17,18]^

**Figure 3:**
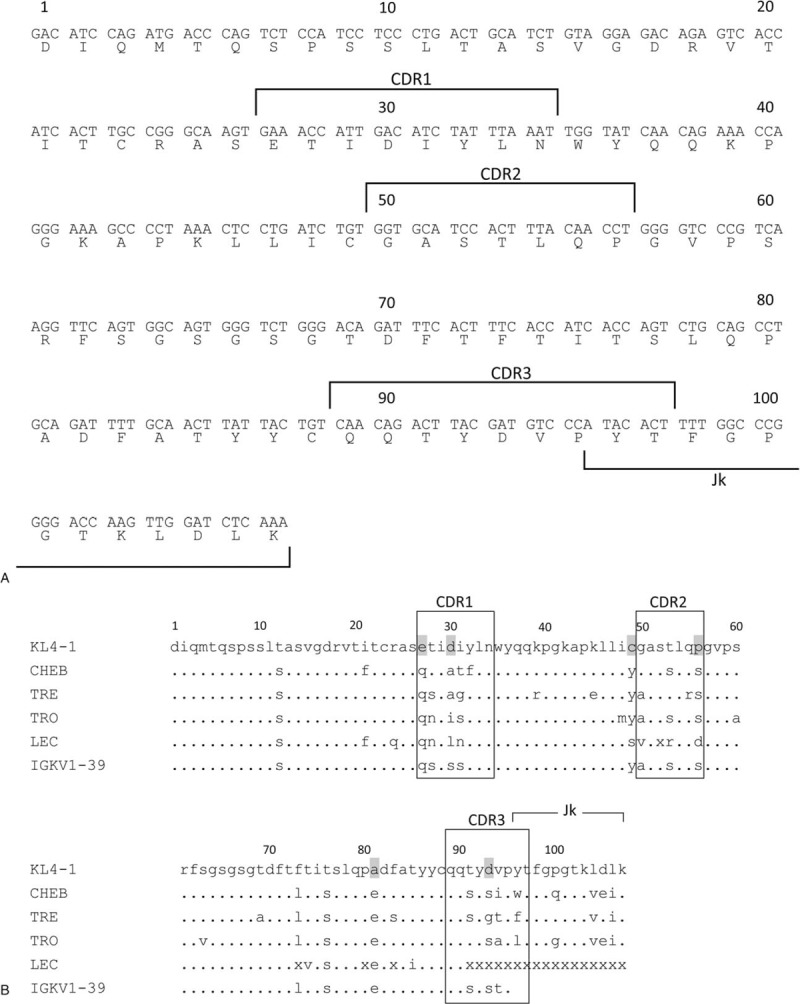
Sequence analysis of the variable region of the present patient with crystalline light chain inclusion-associated kidney disease. (A) Nucleotide and predicted amino acid sequences of V*k* region of clone KL4-1 isolated from the patient. The numbering of amino acid residues in the one-letter code and CDRs are according to Mizuochi et al ^[23]^ The GenBank accession number for KL4-1 cDNA sequence is MH298056. (B) Comparison of the predicted amino acid sequence of clone KL4-1 with those of light chains from previously reported cases of Fanconi syndrome with or without intracellular crystals^[16,20,22]^ and with that of the *IGKV1-39* germ-line gene. Note that the accumulation of needle-like crystals within proximal tubular cells was reported in patients CHEB, TRE and TRO, but not in patient LEC. Unique mutations in our patient are highlighted in grey. Identities are indicated with dots. X: undetermined amino acid residue.

We identified a clone KL4–1 encoding a kappa light chain predominantly expressed in bone marrow. The analysis of nucleotide sequences revealed that its variable region is encoded by the *IGKV1* and *IGKJ2* genes (Fig. 3A) and that the constant region is unmutated and corresponds to the Km(3) allotype. Although none of the known germ-line *IGKV1* gene subgroups were found to be identical to the *KL4-1 Vk* gene, the *IGKV1-39* gene showed the highest homology (91%). The joining region sequence differs from the *IGKJ2* germ-line gene by 4 nucleotide substitutions. The predicted amino acid sequence of the KL4-1 clone differs from that of the *IGKV1-39* and *IGKJ2* germ-line genes by 15 and 3 amino acid substitutions, respectively, including 4 in complementarity-determining region 1 (CDR1), 3 in CDR2 and 3 in CDR3 (Fig. 3B). Among those, we noted the replacement of six unusual amino acid residues: negatively charged glutamic acid for uncharged glutamine at position 27 in CDR1, negatively charged aspartic acid for uncharged serine at position 30 in CDR1, hydrophobic cysteine for tyrosine at position 49 in framework region 2 (FR2), hydrophobic proline for serine at position 56 in CDR2, hydrophobic alanine for charged glutamic acid at position 81 in FR3 and charged aspartic acid for serine at position 93 in CDR3. Significantly, the use of the same *IGKV1-39* was reported for pathogenic monoclonal light chains in all three cases (CHEB, TRE and TRO) of Fanconi syndrome associated with the accumulation of crystals in PTE,^[16]^ while the J*k* gene segment is different: *IGKJ1* for CHEB, *IGKJ3* for TRE and *IGKJ4* for TRO.^[16]^ Notably, amino acid sequences of the V*k* region of CHEB, TRE and TRO are considerably different from that of our case (Fig. 3B). Moreover, none of the above-mentioned unusual amino acid substitutions observed in our case are present in these 3 cases.

## 4 Discussion

We experienced a case showing mild proteinuria and renal insufficiency that was eventually identified as monoclonal gammopathy of undetermined significance (MGUS) with crystalline inclusions within the cells in the kidney. Crystalline inclusions were detected not only within the PTE but also podocytes and histiocytes. Despite the presence of crystalline deposits in the PTE, the patient did not show the manifestations of Fanconi syndrome.

To the best of our knowledge, only 11 cases of crystalline inclusion within the podocytes associated with plasma cell dyscrasia have been reported.^[4,5–15]^ Of these, nine had multiple myeloma,^[5,6,8,9,11–15]^ and two MGUS.^[7,10]^ Interestingly, all cases had IgG-κ M-protein, with only one case exceptionally manifesting clinical manifestations of Fanconi syndrome.^[11]^ Proteinuria is a representative clinical feature of crystalline podocytopathy. About half of the patients had mild proteinuria, while four had nephrotic range proteinuria.^[6,10,12,13]^ Three of these 4 had been diagnosed histopathologically with focal segmental glomerulosclerosis.^[6,10,12]^ Most patients had renal insufficiency of various severities. However, renal glycosuria or hypokalemia was rarely seen in this condition. The clinical and histological features of our case are consistent with those of these previous cases; however, the structure and pathogenicity of monoclonal kappa on podocytes have not been determined in crystal-storing histiocytosis.

It can be difficult to identify crystalline inclusions as monoclonal κ light chains by immunofluorescence. In such cases, immunohistochemical method using paraffin-embedded sections are useful probably because antigenic epitopes are made accessible by anti-κ light chain antibodies through the antigen retrieval procedure.^[4]^ However, in some cases including our case, immunohistochemical method also failed to identify crystalline inclusions as κ light chains. Jones et al speculated that negative immunostaining of crystalline inclusions might be attributable to the inaccessibility of crystalline deposits to anti-κ light chain antibodies owing to their highly crystallized structure.^[19]^ Another possible explanation is partial degradation of the pathogenic light chains. For either of these reasons, we could not identify the crystalline deposits within podocytes as κ light chains in our case.

Previous studies on patients with crystalline light chain inclusion-associated kidney diseases (or myeloma-associated Fanconi syndrome displaying crystals in proximal tubular epithelial cells) revealed that pathogenic monoclonal light chains belong to the germ-line gene of *IGKV1-39* (also known as Vκ1-39 or VκI O2/O12).^[16,20,21]^ Although a number of amino acid substitutions in the V*k* region have been identified,^[16,20,21]^ it has been claimed that the substitution of a polar serine residue by a non-polar hydrophobic amino acid residue, either alanine or isoleucine, at position 30 of CDR1 plays a pathogenic role in the formation of intracellular crystals within tubular cells.^[21,22]^ However, as shown in Fig. 2B, serine at position 30 was also replaced by a hydrophobic leucine in the pathogenic light chain isolated from a patient LEC with Fanconi syndrome but lacking crystalline inclusion in tubular cells.^[22]^ Notably, in our present case, the amino acid residue at position 30 is substituted by a negatively charged aspartic acid. Moreover, we identified 5 other unusual, either hydrophobic or charged, amino acid substitutions not observed in the previously reported cases of LCPT. Of those, the presence of cysteine just next to the CDR2 may be of potential significance because of the possible formation of a unique structure of these light chains through the disulfide-bond formation. If this is proved to be the case, further analysis with its mutants would help define the pathogenic implications of these unusual amino acid residues identified in our case. We have not determined whether this monoclonal light chain sequence is truly pathogenic. Now we are investigating whether the protein encoded by this sequence causes crystalline inclusion in vivo and/or in vitro.

Our present study strongly implicated the *IGKV1-39* gene in crystalline light chain inclusion-associated kidney diseases. However, it needs to be stressed that the V region sequence of our case differs considerably from those of previously reported cases of LCPT that lacked both podocyte involvement and clinically expressed Fanconi syndrome. This could be related to differences in histopathological and clinical manifestations. Clearly, further clonal analyses of patients with light chain crystalline podocytopathy are needed to clarify why despite the use of the same *IGKV1-39* gene for pathogenic light chains, such different histopathological and clinical pictures are induced in these 2 diseases.

## Acknowledgments

We thank John Gelblum for his critical reading of the manuscript and Y. Kubo and M. Kura for their secretarial assistance.

## Author contributions

**Conceptualization:** Mitsuhiro Kawano, Kiyoaki Ito, Satoshi Hara, Kazunori Yamada, Shozo Izui.

**Data curation:** Mitsuhiro Kawano, Kiyoaki Ito, Satoshi Hara, Kazunori Yamada, Takeshi Zoshima, Ichiro Mizushima, Hiroshi Fujii, Ryoichi Miyazaki, Yasukazu Kawai.

**Formal analysis:** Mitsuhiro Kawano, Kiyoaki Ito, Satoshi Hara, Kazunori Yamada.

**Funding acquisition:** Mitsuhiro Kawano, Satoshi Hara, Kazunori Yamada.

**Investigation:** Mitsuhiro Kawano, Kiyoaki Ito, Satoshi Hara, Kazunori Yamada, Takeshi Zoshima, Ichiro Mizushima, Hiroshi Fujii, Yasukazu Kawai, Akihiro Yachie.

**Methodology:** Mitsuhiro Kawano, Kiyoaki Ito, Satoshi Hara, Kazunori Yamada, Takeshi Zoshima, Ryoichi Miyazaki, Akihiro Yachie, Shozo Izui.

**Project administration:** Mitsuhiro Kawano, Kiyoaki Ito, Satoshi Hara, Kazunori Yamada, Shozo Izui.

**Resources:** Mitsuhiro Kawano, Satoshi Hara, Hiroshi Fujii, Ryoichi Miyazaki, Yasukazu Kawai.

**Supervision:** Akihiro Yachie, Shozo Izui, Masakazu Yamagishi.

**Validation:** Mitsuhiro Kawano, Kiyoaki Ito, Satoshi Hara, Shozo Izui, Masakazu Yamagishi.

**Visualization:** Mitsuhiro Kawano, Kiyoaki Ito, Satoshi Hara, Michio Nagata.

**Writing – original draft:** Mitsuhiro Kawano, Kiyoaki Ito, Satoshi Hara, Ichiro Mizushima, Shozo Izui.

**Writing – review & editing:** Mitsuhiro Kawano, Kiyoaki Ito, Satoshi Hara, Kazunori Yamada, Ichiro Mizushima, Michio Nagata, Shozo Izui, Masakazu Yamagishi.
